# Influence of Climatic Variables on Incidence of Whitefly-Transmitted Begomovirus in Soybean and Bean Crops in North-Western Argentina

**DOI:** 10.3390/v15020462

**Published:** 2023-02-07

**Authors:** Pablo Reyna, Franco Suarez, Mónica Balzarini, Patricia Rodriguez Pardina

**Affiliations:** 1 Unidad Ejecutora UFYMA-INTA-CONICET, Córdoba X5020ICA, Argentina; 2 Instituto Nacional de Tecnología Agropecuaria (INTA), Instituto de Patología Vegetal (IPAVE), Av. 11 de Septiembre, Córdoba 4755 X5014MGO, Argentina; 3 Facultad Ciencias Agropecuarias, Universidad Nacional de Córdoba, Córdoba 5000, Argentina

**Keywords:** pathosystem, viral diseases, weather, predictive model

## Abstract

Over the last 20 years, begomoviruses have emerged as devastating pathogens, limiting the production of different crops worldwide. Weather conditions increase vector populations, with negative effects on crop production. In this work we evaluate the relationship between the incidence of begomovirus and weather before and during the crop cycle. Soybean and bean fields from north-western (NW) Argentina were monitored between 2001 and 2018 and classified as moderate (≤50%) or severe (>50%) according to the begomovirus incidence. Bean golden mosaic virus (BGMV) and soybean blistering mosaic virus (SbBMV) were the predominant begomovirus in bean and soybean crops, respectively. Nearly 200 bio-meteorological variables were constructed by summarizing climatic variables in 10-day periods from July to November of each crop year. The studied variables included temperature, precipitation, relative humidity, wind (speed and direction), pressure, cloudiness, and visibility. For bean, high maximum winter temperatures, low spring humidity, and precipitation 10 days before planting correlated with severe incidence. In soybeans, high temperatures in late winter and in the pre-sowing period, and low spring precipitations were found to be good predictors of high incidence of begomovirus. The results suggest that temperature and pre-sowing precipitations can be used to predict the incidence status [predictive accuracy: 80% (bean) and 75% (soybean)]. Thus, these variables can be incorporated in early warning systems for crop management decision-making to reduce the virus impact on bean and soybean crops.

## 1. Introduction

Viral diseases are one of several factors that can affect the yield of legume and grain crops. In Argentina, the most important viral diseases in these crops are caused by begomoviruses. Estimated losses in grain production due to begomoviruses can vary from 40 to 100% depending on the crop, incidence, planting date, and cultivar. Begomoviruses are small, circular, single-stranded DNA viruses (family *Geminiviridae*; genus *Begomovirus*) that infect a wide range of crops in the tropics and subtropics [1]. Epidemics caused by re-emerging and newly emerging geminiviruses are becoming frequent, even in regions that were earlier free from these viruses [2]. The incidence and severity of the diseases caused by begomoviruses have increased considerably in the last two decades. Several crops, such as bean, soybean, tomato, cotton, and cassava are infected with begomoviruses worldwide [3,4].

More than 50 begomoviruses have been detected infecting bean and soybean crops, with bean golden mosaic virus, sida micrantha mosaic virus, tomato severe rugose virus, and mungbean yellow mosaic India virus having been found in both crops [5,6,7]. In Argentina, eight different begomoviruses have been identified: bean golden mosaic virus (BGMV), tomato yellow spot virus (ToYSV), soybean blistering mosaic virus (SbBMV), tomato mottle wrinkle virus (ToMoWrV), sida golden mosaic Brazil virus (SiGMBRV), tomato yellow vein streak virus (ToYVSV), euphorbia mosaic virus (EuMV), and bean bushy stunt virus (BBSV). The former three species have been detected in both bean and soybean crops, whereas ToMoWrV, SiGMBRV, ToYVSV, and BBSV have been found only in bean and EuMV only in soybean crops [8].

Begomoviruses are transmitted by the whitefly *Bemisia tabaci* (Gennadius) (*Hemiptera*: *Aleyrodidae* cryptic species complex) in a persistent manner. Studies have reported that begomovirus transmission follows a density–dependent phenomenon, wherein higher vector occurrence, whitefly populations and/or begomovirus accumulation, in host plants favor rapid virus transmission within crops [9,10]. Presence of whitefly is essential for occurrence of begomovirus outbreaks, since the larger the whitefly populations, the greater the incidence of viruses and the damage they cause [9]. Recent studies suggest that the complex of the whitefly *B. tabaci* comprises many genetically distinct cryptic species [11,12,13], three of which have been detected lately in soybean (New world 2-NW2), bean (NW2, Middle East Asia-Minor 1- MEAM-1 and Mediterranean- MED), tomato (MED), and melon (MEAM-1) in Argentina [14,15]. Because of their broad host range, higher insecticide tolerance, and innate potential, the most invasive members of *B. tabaci*, namely MED and MEAM-1, have largely contributed to the increase of whitefly-mediated direct and indirect damages to crops worldwide [1,16,17,18]. Similarly, in Argentina, since the first report of MEAM-1, this cryptic species has created havoc in broadleaf crops throughout the major agricultural regions of the country, particularly by acting as a vector for begomoviruses [19,20]. Landscapes and weather conditions can significantly affect *B. tabaci* and begomovirus population dynamics. For instance, in uniform farmscapes, a single population of *B. tabaci* can dominate in all cropping systems in the region [21] and dry-warm weather can significantly increase whitefly populations [22]. 

The common bean (*Phaseolus vulgaris* L.) is one of the most important legumes for direct consumption worldwide and a source of protein for millions of people, especially in Latin America, Africa, and the Caribbean [23]. In Argentina, its production in 2019 reached 578,713 tons in a cultivated area of 419,927 ha [24]. Argentina is the world’s leading exporter of Alubia bean and fourth of black, red, and cranberry bean varieties, with such exports accounting for 90% of the country production. Bean is grown in the northwestern (NW) region of Argentina, with Salta province being the main producer, followed by the provinces of Jujuy, Tucumán, Santiago del Estero, Catamarca, and north of Córdoba. The climate in the NW region is humid subtropical with a dry winter season (monsoon regime), with rainfall ranging from 900 to more than 2000 mm a year. 

Argentina is also the world’s third largest producer [24] of soybean (*Glycine max* (L.) Merr), with a mean production of 53 million tons in the last five years [25]. This legume, which is native to China, is used as animal feed and for oil and biodiesel production. Soybean is grown mainly in the Pampas region of Argentina, but the cultivated area is extending to the NW region, where, during the last growing season, 10% of the total area of the country was sown with soybean. In this work, we investigate the relationship between the level of incidence of begomoviruses in bean and soybean crops in the Argentine NW region, where temperatures are relatively high and drought is more frequent. We hypothesized that the relative incidence of begomoviruses in bean and soybean sowed in the NW of Argentina might be influenced by weather conditions and size of vector populations during the pre-sowing period.

## 2. Materials and Methods

### 2.1. Plant Sampling and Analysis

To assess the relative incidence of begomoviruses on soybean and bean crops, plots located in the NW region of Argentina (65.63” and 60.44” W, and 31.34” and 22.49” S) were monitored between 2001 and 2018, with the exception of the 2012 and 2013 growing seasons in which no sample collection was carried out. During this period, more than 2000 symptomatic bean and 1400 soybean samples were collected and maintained at −20 °C until virus analysis. Total nucleic acids of all samples were extracted following the Dellaporta protocol [26] and tested by dot-blot hybridization, with a general probe that detects all begomovirus species. This probe was developed using a mixture of linearized full-length DNA-A clones of BGMV, tomato rugose mosaic virus (ToRMV), and tomato mottle virus (ToMoV), labelled using the DIG DNA Labelling and Detection kit (Roche Applied Sciences). Stringency conditions were adjusted by salt concentration during the washing steps. Membranes were washed twice with 5× SSC and 0.1% SDS at room temperature for 5 min, and twice with 2× SSC and 0.1% SDS at 65 °C for 15 min. All samples that tested positive with the general probe were further analysed with specific probes implemented as the different begomovirus species were identified. The specific probes were obtained by labelling the PCR products corresponding to the highly variable 5’-region of the CP gene. In this case, stringency conditions were as follows: first wash with 2× SSC and 0.1% SDS, second wash 0.5× SSC [27].

### 2.2. Obtaining Biometeorological Variables

Data from 14 climatic variables were extracted using the library “Climate” in R [28] and associated with each sampled field. The extracted climatic variables were: average temperature (AVT), maximum temperature (MXT), minimum temperature (MNT), average dew point temperature (TdAV), average humidity (AVH), minimum average humidity (MNH), maximum average humidity (MXH), wind direction (WDR), wind intensity (Win), atmospheric pressure (ATP), total precipitation (TPP), total cloudiness (TCl), low cloudiness (lCl), and visibility (VIS). To build bio-meteorological variables, data were summarized by adding up precipitation values and averaging other variables in 10-day periods spanning the period before the crop cycle (from July to November). Nearly 200 bio-meteorological variables resulted from multiplying the number of climatic variables by the number of 10-day periods explored per year (14 climatic variables × 14 periods from July to November). 

Each biometeorological variable was denoted with a code consisting of three letters and the number of the month. Data collected over two 10-day periods (20 days) were identified using an underscore with the numbers 1 or 2 to indicate the first or the second 20-day period of the month, respectively. For example, ATP9 = atmospheric pressure of September, ATP9_1 = atmospheric pressure of the first 20 days of September. When 10-day subperiods were considered, they were indicated with the letter *d* plus a number corresponding to the 10-day period starting on the crop planting date, e.g., MXT_d1 = maximum temperature of 10-day period 1, or first 10 days before sowing. A protocol to clean spatial data [29] comprising homologation of spatial coordinates and removal of outliers was applied to the climatic datasets.

#### 2.2.1. Dataset of Begomovirus in Bean Crop

**Figure 1:**
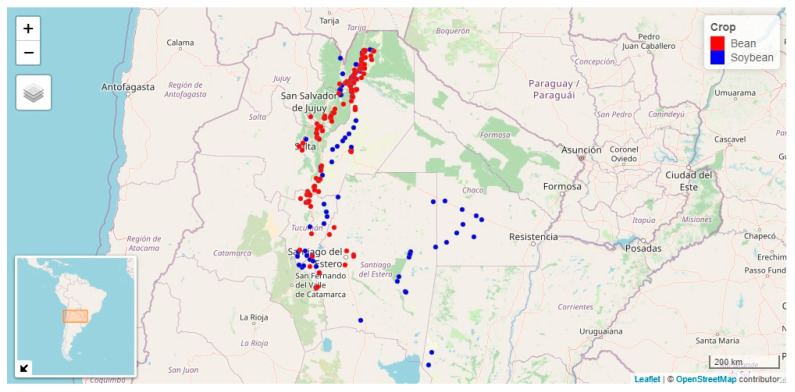
Geographic points with begomovirus incidence in bean (red, *n* = 156) and soybean (blue, *n* = 96) crops in NW Argentina.

The dataset of begomovirus in bean consisted of 1949 records of begomovirus presence or absence obtained between 2001 and 2018, except for 2012 and 2013, in NW Argentina. These data were used to calculate the relative incidence of begomovirus by adding up the positive samples for a geographic point and dividing by the sample size. This procedure resulted in 156 geographic points with disease and climatic data that were further used to explore the climate-incidence relationship (Figure 1). Geographic points with data from a bean plot were classified into plots with moderate (≤50%) or severe (>50%) incidence. A threshold value of 50% was selected, since the average annual relative incidence was 48% and 43% for bean and soybean, respectively. Thus, an incidence value > 50% is above mean for both crops.

#### 2.2.2. Dataset of Begomovirus in Soybean Crop

The soybean dataset included 1242 records collected between 2000 and 2018, except for 2012 and 2013. Relative incidence was calculated as in bean; 96 geographic points were generated for the analysis of the climate-disease relationship (Figure 1). The climatic variables were obtained for the period between mid-September and mid-March. Pre-planting winter months were also considered, with a period of 10 days to summarize the climate data. These variables were then expressed in months; the climatic variables were also obtained every 15 days for September, October, and November.

### 2.3. Statistical Analyses

The biometeorological variables used for modeling relative incidence were obtained using the Boruta machine learning algorithm [30]. This wrapping method was implemented with another ML algorithm (Random Forest [31]) as a regression method. Random forest can capture non-linear relationships and interactions among variables to quantify the importance of each variable in incidence prediction. To fit predictive models, the climatic variables with importance exceeding a given threshold (>10%) were retained. The Boruta algorithm was applied using the package *Boruta* [30] of the software R [32], with the begomovirus incidence categories as the dependent variable. 

Finally, a logistic regression [33] was performed for each pathosystem to explain the categories of begomovirus incidence as a function of the selected biometeorological variables. When the value predicted by the model was >0.5, incidence was predicted to be severe. To further reduce the number of variables in the predictive modeling, a stepwise variable selection method was applied along with the logistic regression model fitting using the variables indicated by the Boruta algorithm as important or tentative. The logistic regression model was fitted with the function *train, method glm* of the package caret [34] in the software R [32]. 

### 2.4. Model Assesement

Different metrics were calculated to evaluate the accuracy of the prediction obtained with the logistic regression model fitted using the selected biometeorological variables. These metrics were calculated using the function *confusionMatrix* of the package caret in R. The sensitivity of the predicted model (i.e., percentage of positive predictive values or of severe incidence that are classified as severe) as well as the specificity (i.e., percentage of values with moderate incidence that are classified as of moderate incidence) were evaluated [35]. The ROC (Receiver Operating Characteristics) curves were used to determine the model discriminant capacity. The ROC curve is the plot of the ratio or proportion of true positives (here called sensitivity) versus the ratio or proportion of false positives. It can also be presented as the ratio of true positives versus 1—ratio of false positives (here called specificity). Among all the summary indices of the ROC curve, the area under the ROC curve (AUC) is the most used by many researchers for evaluation and comparison of models [36]. The discriminative power of each model is related to the corresponding AUC. The larger the area, the better the discrimination ability. AUCs of 0.70–0.79, 0.80–0.89, and >0.90 were used to represent fair, excellent, and outstanding discriminatory ability, respectively [37]. The curves were calculated using the function *accuracy* of the package rfUtilities [38].

### 2.5. Validation of Predictors 

The predictive capacity of the models generated from the logistic regression analysis was tested using cross-validation. This procedure selected 30% of randomly selected records as a validation group. These records were then excluded from the data set used to calibrate the model. This procedure was repeated 30 times, by selecting different random calibration and testing groups. The model fitted for bean was also validated with the datasets from 2019 and 2020; during that period, incidence of begomovirus was monitored in the north of the crop area in Argentina.

## 3. Results

### 3.1. Begomovirus Incidence and Putative Biometeorological Variables

With respect to bean crops, virus analysis using general hybridization probes in all samples resulted in annual incidence values between 16% and 80%, averaging 43%. BGMV was the predominant begomovirus, being present throughout the 16-year period in the bean crops with a relative annual incidence up to 59%. ToYVSV and SbBMV were also present every year but with a lower incidence (up to 13% and 24% for ToYVSV and SbBMV, respectively). Between 2014 and 2016, ToYVSV and ToMOWV were detected, the latter being the predominant begomovirus. SiGMBRV was found from 2015 onwards, but with a very low incidence (1% to 2%).

On the other hand soybean crops, virus analysis using general hybridization probes resulted in annual incidence values of 19% to 96%, averaging 48%. SbBMV was the predominant begomovirus, being present throughout the 16-year period in soybean crops with a relative annual incidence up to 46%. ToYSV and BGMV were also present every year with an average incidence of 13% and 5%, respectively. EuMV was found from 2010 onwards, with an average incidence of 7%.

### 3.2. Selection of Biometeorological Variables

**Figure 2:**
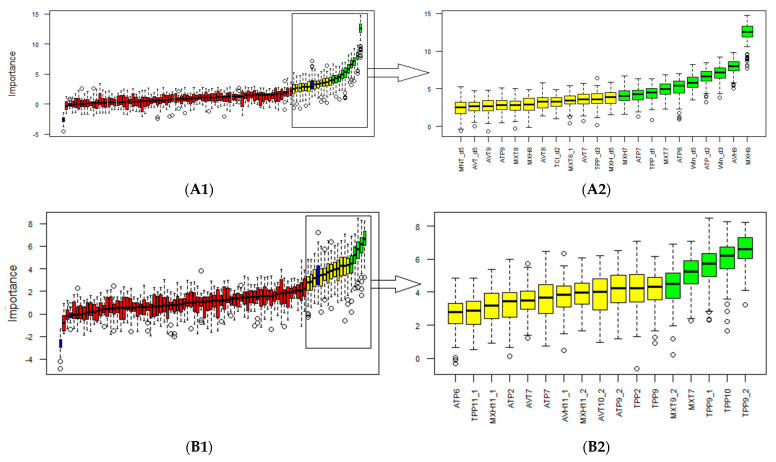
Classification of variables using the Boruta algorithm. Red: Unimportant variables; Yellow: Tentative variables; Green: Important variables. Blue boxes are calculated as reference levels during the run of Boruta algorithm. (**A1**) General classification for beans. (**A2**) Tentative and important variables in beans. (**B1**) General classification for soybeans. (**B2**) Tentative and important variables in soybeans.

The variable selection procedure for beans identified 10 variables as important and 12 as tentative; the remaining ones were of low importance (Figure 2A1,A2). For soybeans, the Boruta algorithm classified five variables as important and 12 as tentative (Figure 2B1,B2).

### 3.3. Regression Models Fitted to Predict Begomovirus Incidence

In the case of beans, the fitted logistic regression [Accuracy: 0.80, Sensitivity: 71%, Specificity: 86%] modeled the logarithm of the chances of severe incidence based on the variables maximum temperature in the first 10-day period of June (MXT6_1), maximum temperature in July (MXT7), mean temperature in July (AVT7), maximum temperature in August (MXT8), mean temperature in August (AVT8), average humidity in September (AVH9), and total precipitations in the first 10-day period (TPP_d1) before planting date (Table 1). The probability of high incidence of begomovirus (>50%) derived from the fitted model is:

(1) $$\frac{exp^{9.07-0.36\;MXT6\_1+2.14\;MXT7-2.32AVT7-2.20\;MXT8+3.64\;AVT8+-0.42\;AVH9-0.015\;TPP\_d1}}{1+exp^{9.07-0.36\;MXT6\_1+2.14\;MXT7-2.32AVT7-2.20\;MXT8+3.64\;AVT8+-0.42\;AVH9-0.015\;TPP\_d1}}$$

For the begomovirus pathosystem in soybeans, the fitted predictive model [Accuracy: 75%, Sensitivity: 72%, Specificity: 89%] included the following climatic variables of high predictive capacity: mean temperatures in the first 20 days of September (AVT9_1), total precipitation in the last 20 days of September (TPP9_2), and mean temperature in the last 20 days of October (AVT10_2) (Table 1). The probability of high incidence (>50%) of begomovirus derived from the fitted model is:

(2) $$\frac{exp^{10.05+0.30\;AVT9\_1-0.16\;TPP9\_2-0.62\;AVT10\_2}}{1+exp^{10.05+0.30\;AVT9\_1-0.16\;TPP9\_2-0.62\;AVT10\_2}}$$

Therefore, a higher number of variables were needed to explain the relative incidence of begomovirus in beans than in soybeans. In beans, data not only on average temperatures but also on maximum temperatures were needed. The most relevant temperature values were those of July and August, i.e., periods prior to those selected for the soybean model (September–October).

**Figure 3:**
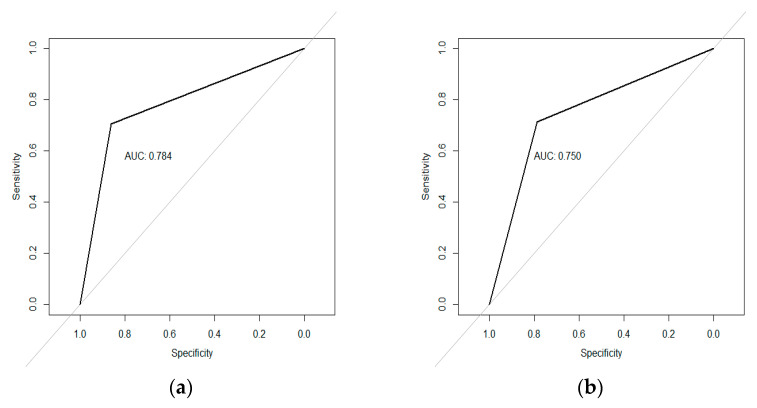
ROC curve and area under the curve (AUC). (**a**) Beans. (**b**) Soybeans.

The associated ROC curves showing good capacity to differentiate sites with high probability of severe incidence from those with high probability of moderate incidence are presented in Figure 3. The bean model was evaluated with 10 new records of begomovirus incidence from the north of Argentina after downloading the climatic variables of the 2019–2020 crop season; classification was correct in 90% of the cases.

## 4. Discussion

The main cause of occurrence of most plant viruses is anthropogenic introduction of parasites, although severe weather events are also important drivers of disease emergence [39]. Emerging virus diseases, like those caused by geminiviruses in plant systems, is challenging food production in tropical and subtropical areas, where high temperatures and drought periods are frequent. Geographic distribution of plant diseases is largely related to the environmental conditions prevailing in the crop area. Begomovirus diseases cause economic losses through the reduction of grain quantity and quality, and the increase in production costs from the use of agrochemicals or seeds bred for resistance to pathogens [40,41,42].

Both pathosystems analyzed in this work include begomovirus, an agent associated with important yield losses in the NW subtropical area of Argentina, where beans are produced and soybeans are increasingly cultivated. The results of our study suggest that maximum winter temperature, relative humidity in September, and precipitations 10 days before planting date are variables with high capacity to predict the level of begomovirus incidence in bean crops. Similarly, for soybeans, our findings indicate that mean temperatures in September and October and total precipitation in the last 20 days of September are the variables that best explained begomovirus incidence, since soybeans are commonly planted in September and October in NW Argentina. 

It is known that both distribution and incidence of numerous viruses are directly associated with the distribution and population dynamics of the insect vector [14,43,44]. According to Morales et al., begomoviruses largely depend on the weather conditions that favor whitefly reproduction and survival [9]. Prolonged warmer and dry weather conditions often favor higher whitefly populations and begomovirus incidence [16].

In agreement with other works, the models fitted in this work indicate an increase in begomovirus incidence in these crops when pre-planting months are warm. Krause-Sakate et al., 2020 [16] suggest that whitefly populations are affected by extreme low temperatures, low relative humidity (<60%), and persistent precipitation. It is well known that whiteflies do not adapt to elevations higher than 1000 m asl due to climatic limitations. Temperature is one of the most critical variables for the life development of the insect vector, influencing colonization, behavior, and distribution [16]. In general, *B. tabaci* incidence is higher in regions with a dry season lasting at least four months, precipitation records below 80 mm during that season, and a monthly mean temperature of at least 21 °C in the warmest months of the year [45]. As global warming increases, so does the probability of the occurrence of these weather conditions in bean and soybean subtropical crop areas, like those in NW Argentina [46].

On average, the vector life cycle ranges between 17 and 27 days, but it can be completed over 12–14 to 43–49 days, depending on warm or cool weather conditions, respectively [47]. The temperature range for development is very wide, with 16 and 24 °C being the most favorable temperatures. Temperatures below 9 °C and above 40 °C are lethal to the insect. Regarding relative humidity, optimum values between 30% and 60% have been reported [48,49,50]. In the study area, relative humidity ranges between 35% and 65%; values close to the maximum values of the relative humidity range are recorded in spring and were negatively associated with virus incidence. 

Information about geminivirus and climate is abundant, but few works have developed predictive models of incidence based on weather conditions, suggesting that the impact of biological correlations on management is underestimated. The models fitted using the begomovirus-weather relationship in this work show high predictive capacity of severe incidence of the virus. Since the variables with greatest contribution are recorded in pre-planting periods, the models could be integrated into warning systems that also consider other factors, such as crop susceptibility, availability of alternative hosts that allow vector feeding and reproduction, and agronomic management of the affected plots. The inclusion of predictive models in early warning systems for decision-making has helped reduce the risk of virus infection in other crops [51]. Nevertheless, this type of predictive models should be used taking into account that the prediction of the probability of disease development in a crop is characteristic of and exclusive to each pathosystem and can be influenced by other factors not included in the model. 

## 5. Conclusions

This work demonstrated that biometeorological variables are correlated with the level of begomovirus incidence in bean and soybean crops. In addition, those variables can be used to generate warning systems that allow producers to implement agronomic practices, such as selection of varieties and sowing dates, to reduce the effect of the virus on bean and soybean crops. For beans, high maximum temperatures in winter, low humidity in spring, and precipitation 10 days before planting are correlated with severe incidence. In soybeans, high temperatures in late winter and before planting and low precipitation in spring were good predictors of high begomovirus incidence. The information in the manuscript has practical applications in terms of disease management in NW Argentina.

## Figures and Tables

**Table 1:** Regression coefficients that explain the logarithm of the chances of severe incidence of begomovirus in beans and soybeans cultivated in NW Argentina.

		Estimate	Std. Error	z Value	Pr(>|z|)	
Bean	Intercept	9.0749	10.0292	0.905	0.365545	
	MXT6_1	−0.3599	0.1233	−2.920	0.003506	**
	MXT7	2.1496	0.7224	2.976	0.002925	**
	AVT7	−2.3241	0.6961	−3.338	0.000842	***
	MXT8	−2.1989	0.5513	−3.989	6.64 × 10^−5^	***
	AVT8	3.6146	0.8607	4.200	2.67 × 10^−5^	***
	AVH9	−0.4208	0.0998	−4.213	2.52 × 10^−5^	***
	TPP_d1	−0.0152	0.0071	−2.132	0.033041	*
Soybean	Intercept	10.050	4.918	2.044	0.040994	*
	AVT9_1	0.303	0.123	2.456	0.014043	*
	TPP9_2	−0.160	0.047	−3.376	0.000734	***
	AVT10_2	−0.622	0.231	−2.691	0.007125	**

Meaning of symbols: ‘***’:0; ‘**’: 0.001; ‘*’: 0.05. MXT6_1: maximum temperature in the first 10-day period of June, MXT7: maximum temperature in July, AVT7: mean temperature in July, MXT8: maximum temperature in August, AVT8: mean temperature in August, AVH9: average humidity in September, TPP_d1: total precipitations in the first 10-day period before sowing date, AVT9_1: mean temperatures in the first 20 days of September, TPP9_2: total precipitations in the last 20 days of September, AVT10_2: mean temperature in the last 20 days of October.

## Data Availability

Not applicable.
